# Vitamin E nanoparticles enhance performance and immune status of Nile tilapia

**DOI:** 10.1186/s12917-024-04398-w

**Published:** 2024-12-12

**Authors:** Enas A. H. Farag, Mohamed Z. Baromh, Naglaa El-kalamwi, Ahmed H. Sherif

**Affiliations:** 1 Department of Pharmacology, Animal Health Research Institute AHRI, Agriculture Research Center ARC, Benha, Egypt; 2 Division of Aquaculture, National Institute of Oceanography and Fisheries (NIOF), Alexandria, Egypt; 3 Pathology Department, Animal Health Research Institute AHRI, Agriculture Research Center ARC, Dokki, Egypt; 4 Fish Diseases Department, Animal Health Research Institute AHRI, Agriculture Research Centre ARC, Kafrelsheikh, Egypt

**Keywords:** *Oreochromis niloticus*, Growth, Phagocytic activity, Lysozyme, Tocopherol, Chitosan, Nanoparticles

## Abstract

Vitamin E (VE) is an essential vitamin liposoluble antioxidant in aquatic animals that is usually lost during feed processing and digestion, whereas nano-chitosan, a polysaccharide, could protect VE. In this study, Nile tilapia (70.85 ± 0.2 g) was fed VE (100 mg/kg dry diet) and a chitosan protected-VE nanoparticle (NPs) with gradual percentages of recommended dose 25%, 50%, 75%, and 100% for 4, 6, and 8 weeks. Growth parameters total weight gain (TG), daily weight gain (DWG), and relative growth rate (RGR) were significantly and positively correlated with VENPs additions. Regardless of the addition level, the feed conversion ratio (FCR) was significantly lower in the VENP groups. Lysozyme, serum antibacterial activity, and oxidative burst activity indicated the superiority of VENPs (VENPs75 and VENPs100) in enhancing the fish’s innate immunity compared to bulk VE and the control groups. Fish were experimentally challenged with pathogenic *Aeromonas hydrophila;* those received dietary showed a low mortality rate (MR%), about 40% compared with 70% in the control with lower re-isolation compared to the control and VE groups. VENPs could provide ascending relative protection level during the period of 4 to 8 weeks; RPL ranged from 33.3 to 42.86% (VENPs100), 16.67–42.86% (VENPs75), 0 to 28.57% (VENPs50), and 0 to 14.29% (VENPs25 and VE), respectively. Finally, this study recommended incorporating VENPs into the Nile tilapia diet at 50, 75, and 100 mg/ kg fish feed. Fish in the VENPs75 and VENPs100 groups were immune boosted, becoming less vulnerable to A. hydrophila infection.

## Introduction

Investments rapidly and constantly grow in Egyptian aquaculture as net farmed fish production rose from 139,389 tons to 1,561,457 tons between 1998 and 2018 (FAO 2003–2020), becoming a source of hard currency and food security; nowadays, Egypt is considered one of the top fish producers in Africa and is ranked third worldwide in Nile tilapia production after Indonesia and China [1, 2]. Epidemic diseases usually accompany intensive animal production, and scientists seek alternative and effective treatments besides traditional and approved medicines, such as metal nanoparticles, nanocomposites, nanocapsules, and conjugates that are biocompatible with aquatic animals’ environments [3–6].

Nanotechnology is used to produce some nanomaterials applied in the aquatic industry, mainly feed additives, nonmetals (selenium, iron, copper, and silver), and nano-sized chitosan used as a coat for some medicines [7]. Some nanomaterials could counteract bacterial infection by interacting with the bacterial cell walls and evacuating cell content; their size provides high availability as they can easily cross tissues and remain more extended than the bulk form. Finally, it could stimulate fish immunity [8–16]. Many aquatic studies showed that nano-sized chitosan (biopolymer polysaccharide) has antibacterial and immunostimulant properties, providing a high relative level of protection for aquatic animals, and is considered an environmentally friendly product [17–19].

Many natural products were used to increase the antioxidant capacity of aquatic animals [20–22]. However, vitamin E (a liposoluble vitamin) is the most reliable antioxidant and immunostimulant agent. It inhibits lipid peroxidation and protects animal cells against generated reactive oxygen species (ROS) [23, 24] by binding to lipid free radicals using phenolic hydrogen [25]. Applying a regular dose of VE can promote animal performance and regulate the composition of fatty acids [26].

So, the present work highlighted the role of dietary nano-vitamin E (tocopherol alpha), which is protected with nano-chitosan, as a booster agent of growth and immunity for Nile tilapia fish *(Oreochromis niloticus).* Adding vitamin E nanoparticles (VENPs), protected with nano-chitosan, could help fish become invulnerable to *Aeromonas hydrophila* infection.

## Materials and methods

### Fish husbandry

A two hundred and seventy Nile tilapia *(Oreochromis niloticus)*, weighing about 70.85 ± 0.2 g, were purchased from a freshwater fish farm in the north of Egypt. Fish were tranquillized (40 mg MS222 /L, SyncaineR, Syndel, Canada) [27] and immediately shipped to the fish unit of the Animal Health Research Institute (AHRI). Upon arrival, fish were disinfected using 10 ppm iodine for 10 s (povidone-iodine 5%, Nile Company for Pharmaceuticals, Egypt) [28]. Fish were stocked into a water tank (2 × 1.5 × 1 m) during the 14 days of acclimatization where water parameters were temperature (27.5 ± 0.5 °C), pH (7.9 ± 0.1), and salinity (0.48 ± 0.1 g/L). One-third of tank water was replaced with clean, de-chlorinated water. Fish were fed twice daily at a rate of 5% of their live body weight, at 10:30 a.m. and 04:00 p.m. The chemical composition of fish feed was crude protein (CP) 30.28%, digestible energy (DE) 2980 kcal/kg. Crude fibre 2.5%, Nitrogen-free extract (NFE) 36.1.3%, and Ash 7.14%). For sampling, fish was tranquillized by immersion in 40 mg (Tricaine methanesulfonate MS-222, SyncaineR, Syndel, Canada) /L water. For tissue sampling, the experimental fish was euthanized by immersion in 250 mg MS-222 /L water /10 minutes.

### Feeding trial

In the feeding trial with different additive levels, the recommended dose of VE is 100 mg/kg dry diet for Nile tilapia [29]. Experimental fish fed on gradual doses of VENPs (25%, 50%, 75%, and 100% of the recommended dose), for 4, 6, and 8 weeks. Growth performance was calculated using the equations listed below:

$$\:\text{T}\text{o}\text{t}\text{a}\text{l}\:\text{w}\text{e}\text{i}\text{g}\text{h}\text{t}\:\text{g}\text{a}\text{i}\text{n}\:\left(\text{T}\text{G}\right)=\text{F}\text{i}\text{n}\text{a}\text{l}\:\text{b}\text{o}\text{d}\text{y}\:\text{w}\text{e}\text{i}\text{g}\text{h}\text{t}\:\left(\text{g}\right)-\text{I}\text{n}\text{i}\text{t}\text{i}\text{a}\text{l}\:\text{b}\text{o}\text{d}\text{y}\:\text{w}\text{e}\text{i}\text{g}\text{h}\text{t}\:\left(\text{g}\right)\:$$

$$\:\text{W}\text{e}\text{i}\text{g}\text{h}\text{t}\:\text{g}\text{a}\text{i}\text{n}\:\left(\text{W}\text{G}\right)\text{\%}=\frac{\text{T}\text{G}}{\text{I}\text{W}}\:\times\:100$$

$$\:\text{D}\text{a}\text{i}\text{l}\text{y}\:\text{w}\text{e}\text{i}\text{g}\text{h}\text{t}\:\text{g}\text{a}\text{i}\text{n}\:\left(\text{D}\text{W}\text{G}\right)=\frac{\text{T}\text{G}}{\text{t}\text{i}\text{m}\text{e}\:\left(\text{d}\text{a}\text{y}\text{s}\right)}\:$$

$$\:\text{R}\text{e}\text{l}\text{a}\text{t}\text{i}\text{v}\text{e}\:\text{G}\text{r}\text{o}\text{w}\text{t}\text{h}\:\text{R}\text{a}\text{t}\text{e}\:\left(\text{R}\text{G}\text{R}\right)\:\text{\%}=\frac{\text{F}\text{W}\--\text{I}\text{W}}{\text{I}\text{W}\:}\:\times\:100$$

$$\:\text{F}\text{e}\text{e}\text{d}\:\text{c}\text{o}\text{n}\text{v}\text{e}\text{r}\text{s}\text{i}\text{o}\text{n}\:\text{r}\text{a}\text{t}\text{i}\text{o}\:\left(\text{F}\text{C}\text{R}\right)=\frac{\text{F}\text{e}\text{e}\text{d}\:\text{i}\text{n}\text{t}\text{a}\text{k}\text{e}\:\left(\text{F}\text{I}\right)}{\text{T}\text{G}}$$

### Syntheses of vitamine E and chitosan nanoparticles (ionotropic-gelation method)

Nanocomposite was formed using the interaction between the tripolyphosphate group (negative charge) and molecules of the chitosan amino group (positive charge) [30]. The formed nanoproduct was characterised by high-resolution transmission electron microscopy (TEM-JEM1400F HRTEM) using 300 keV beam energy. Nanocomposite was added to fish feed: feed pellets were soaked in water and blended, then nanoparticles were added and mixed with gelatin 5% w/w. All chemicals were obtained from the local market.

- Chitosan (Sigma-Aldrich, USA), molecular weight (MW) of 50–90 kDA, de-acetylation degree ≥ 75%.
- Vitamin E, DL-alpha-Tocopherol acetate (Glentham Life Science England).
- Gelatin (Nutri-B-Gel) produced by Canal Aqua Cure, Egypt.

### Innate immunity

#### Oxidative burst activity (OBA)

The OBA of fish heterophils was performed using a nitroblue tetrazolium (NBT) stain (Fluka Buchs, Switzerland) [31]. A drop of fish blood was placed on a cover slide and stained with 50 µl of NBT (0.2%). The stained cover slide was kept at room temperature (27.5 ± 0.5 °C) for 30 min and then gently washed with PBS (pH 7.4). The stuck cells and stained dark blue were counted under the light microscope.

#### Serum antibacterial activity (SAA) [32]

The test was performed by mixing 100 µl experimental fish serum and 100 µl bacterial suspension (*Aeromonas hydrophila*, 2 × 10^8^ CFU). The mixture was aerobically incubated at 25 °C/1 h, then inoculated onto nutrient agar plates. The grown colonies, which represented the viable bacterial cells, were counted. The percentage of dead cells to the initial bacterial count was determined.

#### Analysis of lysozyme activity (LYZ) [33]

Lysozyme activity was assayed using the enzyme-linked immunosorbent assay (ELIZA). Fish serum (10 µL) was mixed with *Micrococcus lysodeikticus* (100 µL) and PBS (360 µg/mL, pH:6.24). The mixture was incubated for 5 min at 37 °C, and the sample was measured at 450 nm absorbance.

### Microbial challenge and re-isolation

To determine the impact of dietary-VENPs on fish performance, the experimental Nile tilapia was challenged against pathogenic strain of *A. hydrophila* (AHRAS2-accession number GenBank MW092007) [34]. A Fifteen fish were intraperitoneally injected with 2.4 × 105 CFU/mL (LD50) suspended in 0.1 mL normal saline (0.65%). The bacterial strain was re-isolated for all injected fish. Twenty fish of the negative control was injected (I/P) with 0.1 mL of normal saline [35]. For fourteen days of observation, the number of dead fish was estimated, and the mortality rate (MR %) and the relative protection levels (RLP) [36] provided by VENPs were determined following the equations below:

$$\:\text{M}\text{R}\left(\text{\%}\right)\:=\frac{\text{n}\text{u}\text{m}\text{b}\text{e}\text{r}\:\text{o}\text{f}\:\text{d}\text{e}\text{a}\text{d}\:\text{f}\text{i}\text{s}\text{h}\:\:}{\text{f}\text{i}\text{s}\text{h}\:\text{p}\text{o}\text{p}\text{u}\text{l}\text{a}\text{t}\text{i}\text{o}\text{n}}\times\:\:100$$

$$\:\text{R}\text{L}\text{P}\text{\%}=(1-\frac{\text{\%}\:\text{d}\text{e}\text{a}\text{d}\:\text{o}\text{f}\:\text{t}\text{r}\text{e}\text{a}\text{t}\text{e}\text{d}\:\text{f}\text{i}\text{s}\text{h}\:}{\text{\%}\:\text{d}\text{e}\text{a}\text{d}\:\text{o}\text{f}\:\text{c}\text{o}\text{n}\text{t}\text{r}\text{o}\text{l}\:\text{f}\text{i}\text{s}\text{h}})\times\:\:100$$

### Statistical tests

The performance of Nile tilapia supplemented with different levels of dietary VENPs was estimated using two-way ANOVA (SPSS software version 22). The obtained data was analyzed to determine the mean and standard error (SE), and the significance of the difference was calculated using Duncan’s test at *P* ≤ 0.05.

## Results

### Specification of manufactured vitamin E nanoparticles (VENPs)

**Fig. 1:**
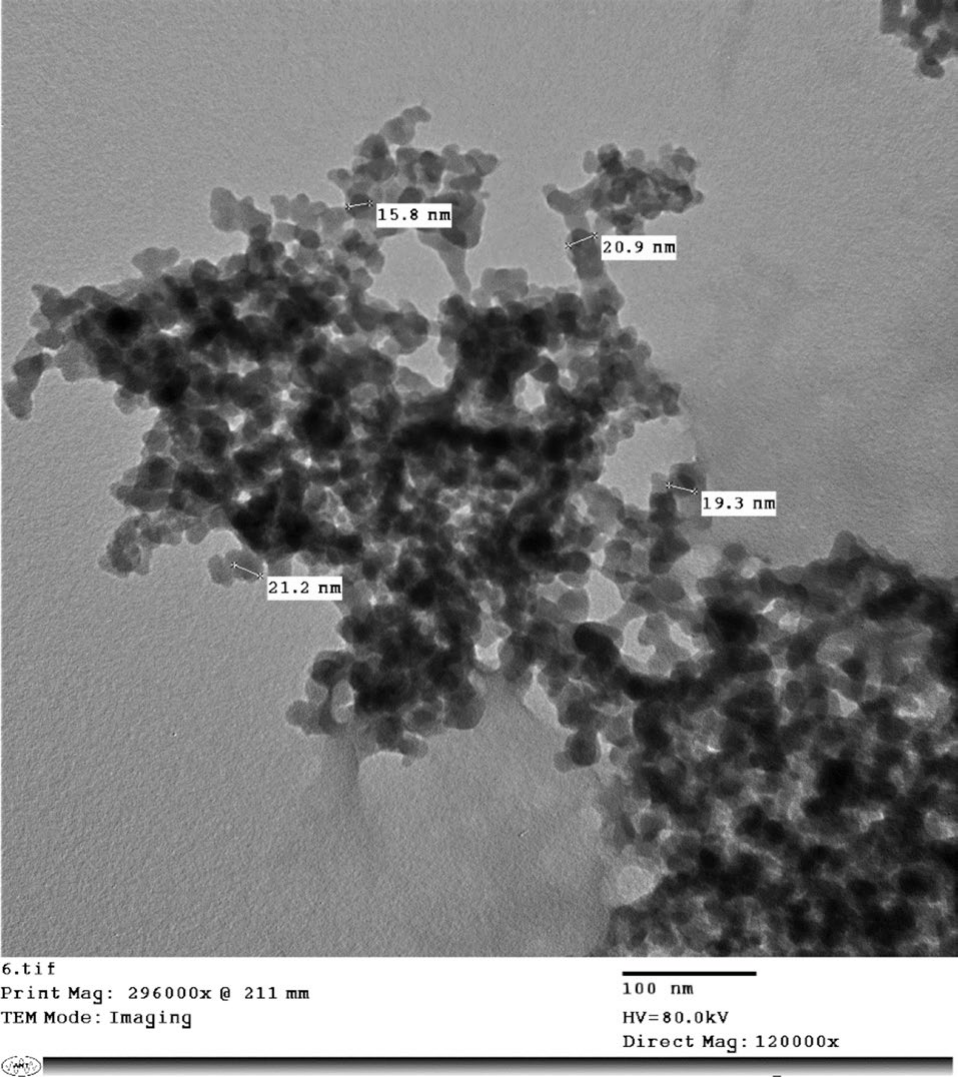
Nanocomposite VENPs characterization of the formed using the transmission electron microscope, TEM

The prepared VENPs were photographed using a transmission electron microscope (TEM), and the size of the nanocomposite was about 20 nm. Each 1 gram contained 100 mg of VE protected with chitosan (Fig. 1).

### Fish growth performance

This is a comparison between VE, a recommended dose of 100 mg/kg dry diet, and VENPs, with gradual percentages of the recommended dose of 25%, 50%, 75%, and 100%.

**Fig. 2:**
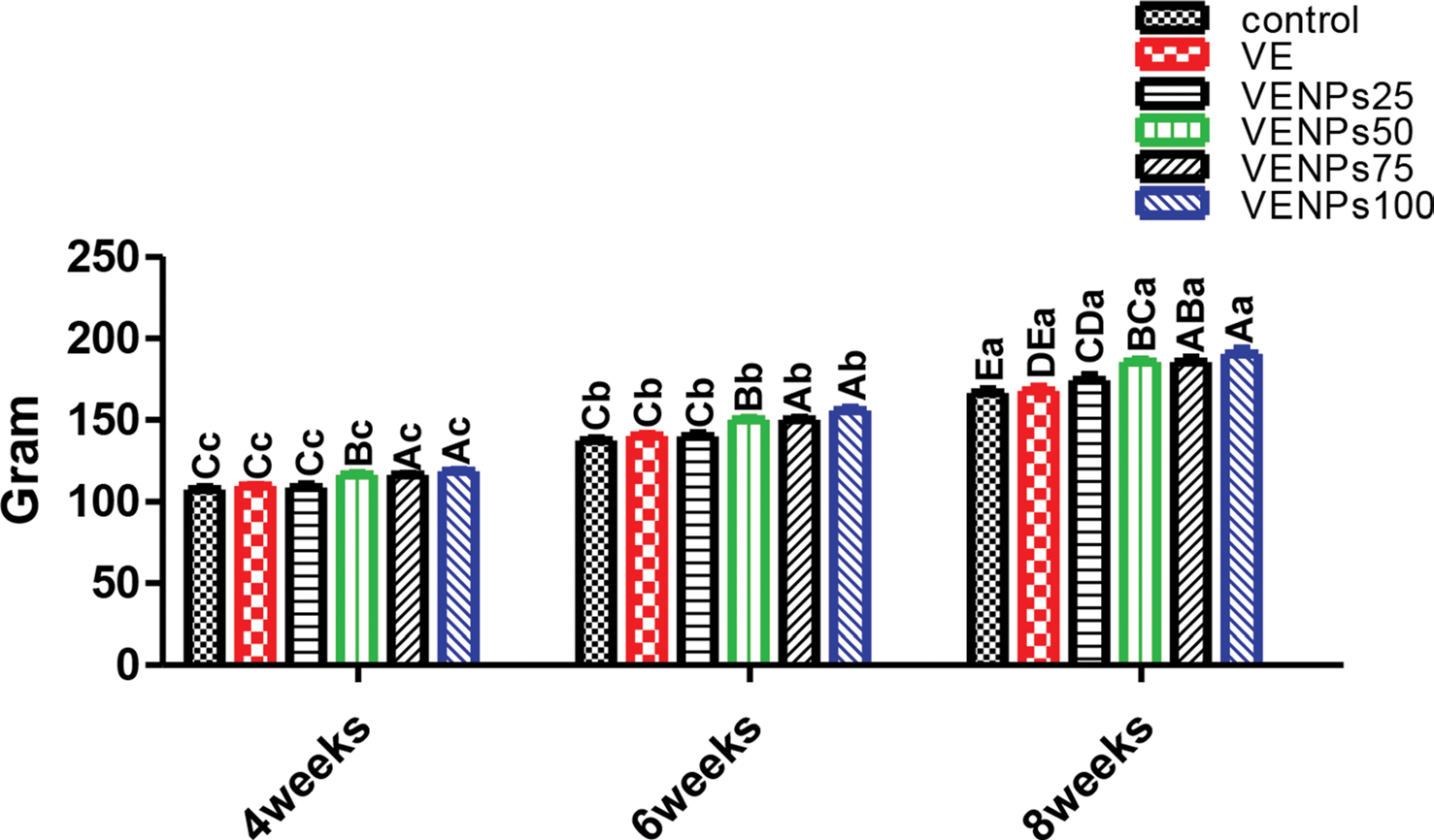
Final weight (FW) (g/fish) of the experimental Nile tilapia. Notes: VE; vitamin E, VENPs; vitamin E nanoparticles (25, 50, 20, and 100 mg/kg fish feed). Data as means (column) and standard error (small bar), values with different letters differ are significantly differed at (*P* ≤ 0.05) capital one (between the treatments in same period) small one (the same treatment in different periods)

In Fig. 2, FW was insignificantly different between VE and VENPs 25% (109.4 and 108.65 g) in the 4-week feeding trial compared to the control 107.38 g. The same trend was achieved in the 6-week and 8-week feeding trials. High doses of nanocomposite VENPs50, VENPs75, and VENPs 100 had significantly higher values regardless of feeding periods.

**Fig. 3:**
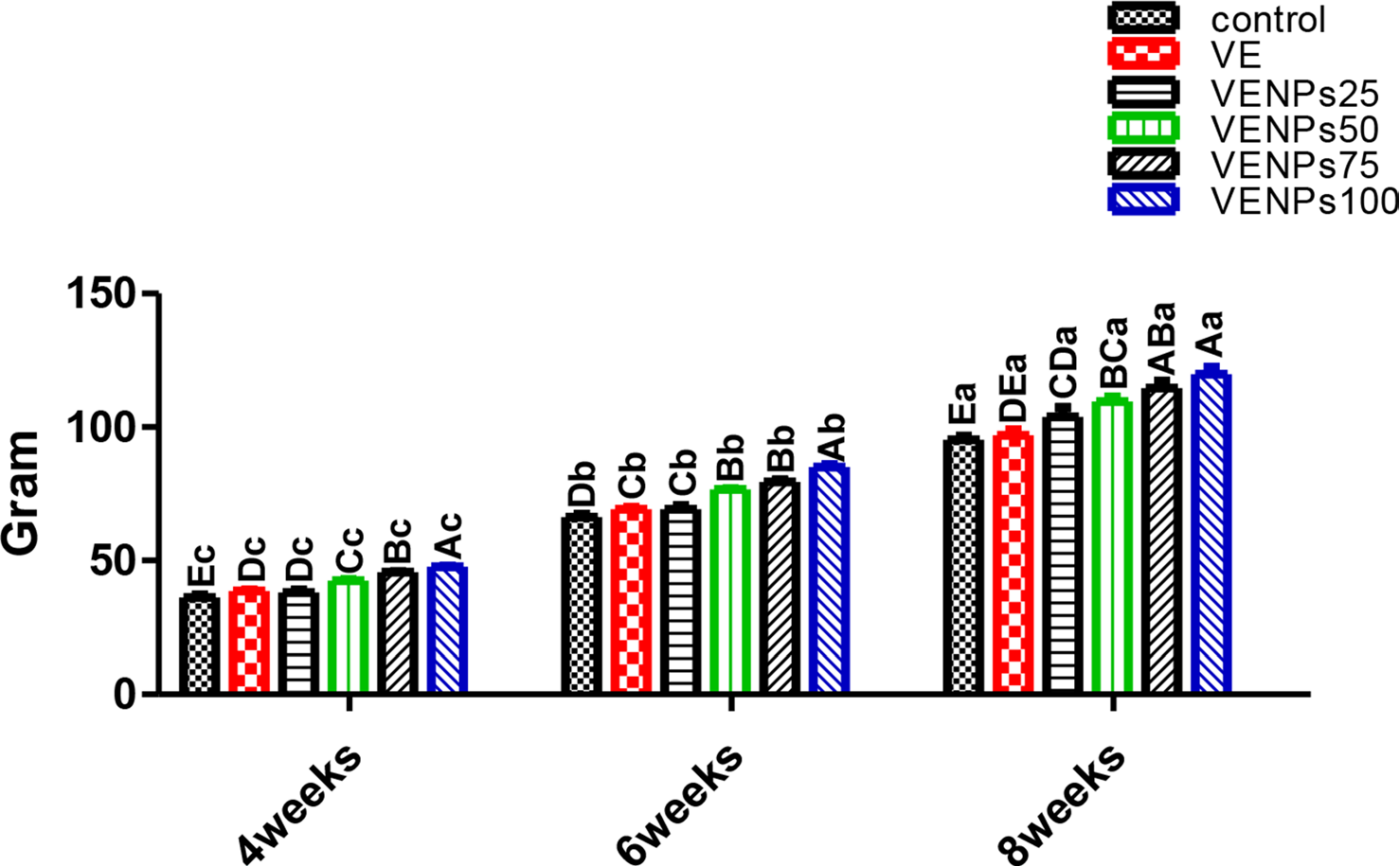
Total gain TG (g/fish) of the experimental Nile tilapia. Notes: VE; vitamin E, VENPs; vitamin E nanoparticles (25, 50, 20, and 100 mg/kg fish feed). Data as means (column) and standard error (small bar), values with different letters differ are significantly differed at (*P* ≤ 0.05) capital one (between the treatments in same period) small one (the same treatment in different periods)

**Fig. 4:**
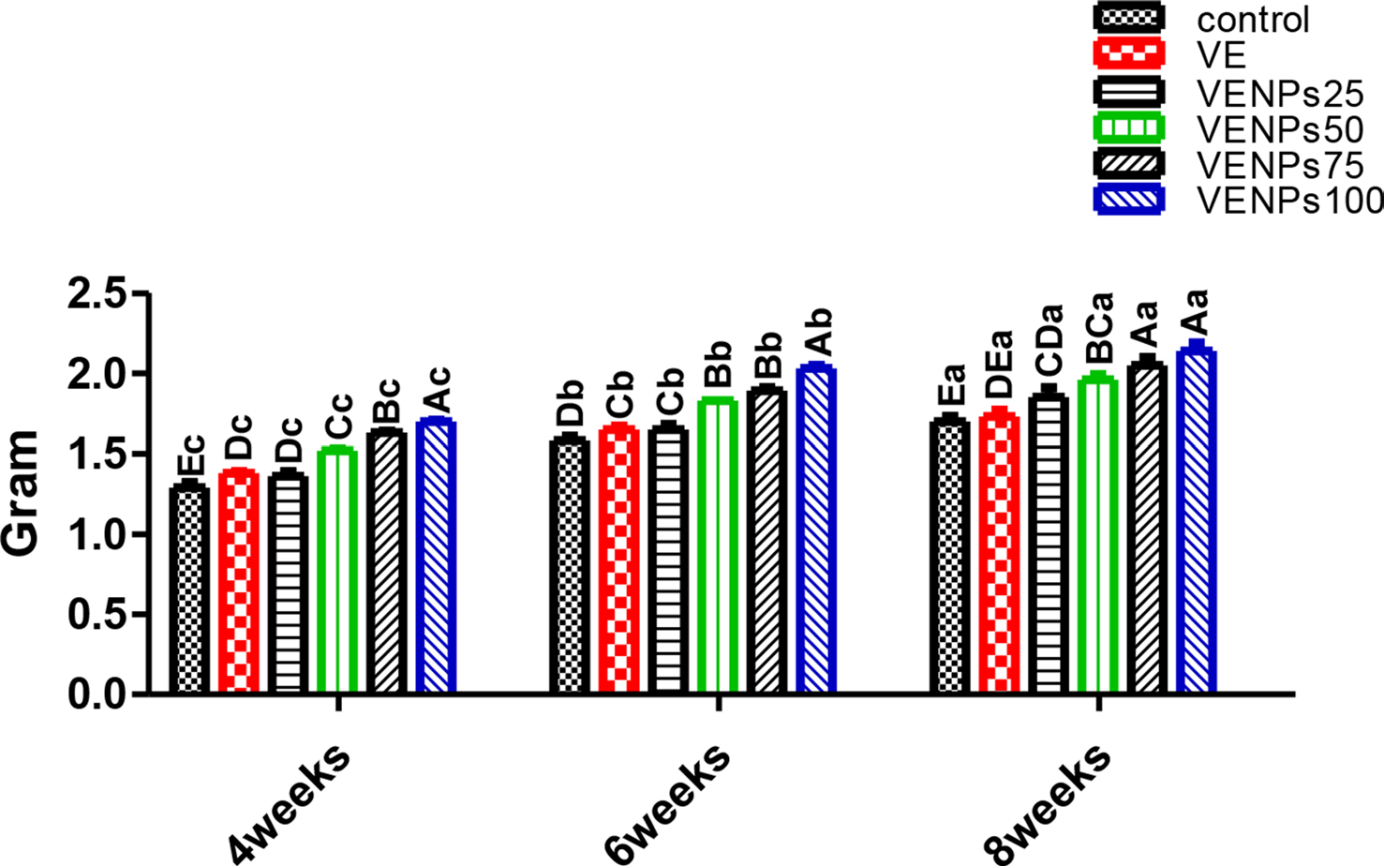
Daily weight gain (DWG) (g/fish) of the experimental Nile tilapia. Notes: VE; vitamin E, VENPs; vitamin E nanoparticles (25, 50, 20, and 100 mg/kg fish feed). Data as means (column) and standard error (small bar), values with different letters differ are significantly differed at (*P* ≤ 0.05) capital one (between the treatments in same period) small one (the same treatment in different periods)

In Fig. 3, TG was significantly and gradually raised with increasing incorporation of VENPs, with an insignificant difference between VE and low VENPs (25%). Also, it was noticed that after the 8-week feeding trial, VENPs 25% had superiority over VE 103.8 and 96.88 g, respectively; compared to the control 95.2 g. DWG (Fig. 4) had the same trend as TG, as VENPs had superiority with gradual enhancements by increasing the level of incorporations.

**Fig. 5:**
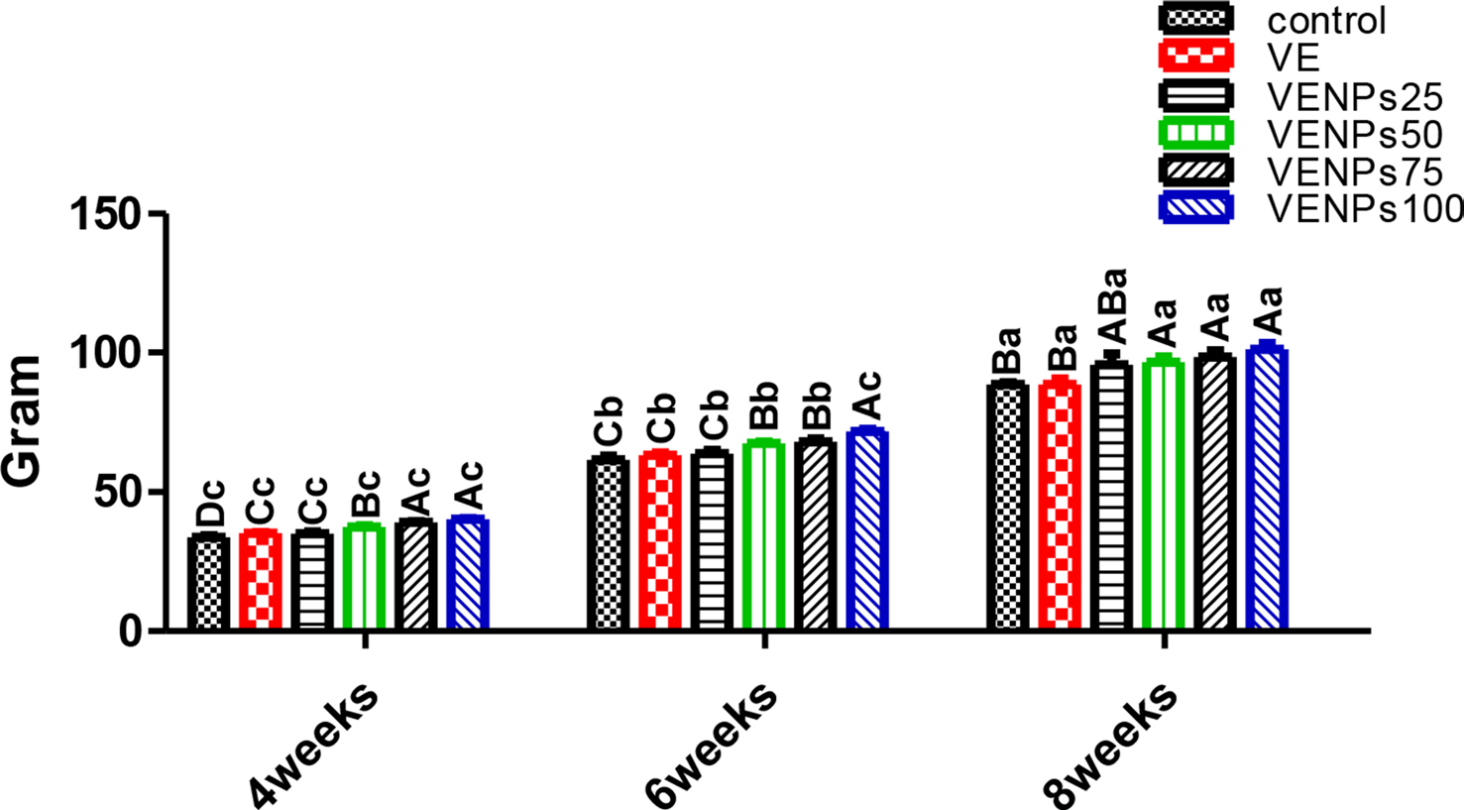
Relative growth rate (RGR) % of the experimental Nile tilapia. Notes: VE; vitamin E, VENPs; vitamin E nanoparticles (25, 50, 20, and 100 mg/kg fish feed). Data as means (column) and standard error (small bar), values with different letters differ are significantly differed at (*P* ≤ 0.05) capital one (between the treatments in same period) small one (the same treatment in different periods)

Regardless of the feeding period, RGR (Fig. 5) was higher in VENP groups with linear increase with elevation incorporation percentages. After 4 weeks of feeding, both VENPS 100 and 75 had significantly higher RGR, followed by VENPs50, 40.28%, 39.2%, and 37.5%, respectively. VENPs25 and VE differed significantly higher than the control, 34.95%, 35.23, and 33.72%, respectively. RGR after the 6-week feeding trial had the same trend as the 4-week. After 8 weeks, there was no significant difference between VENP groups, which were significantly improved compared to fish that received VE (bulk form) and the control ones (*P* ≤ 0.05).

**Fig. 6:**
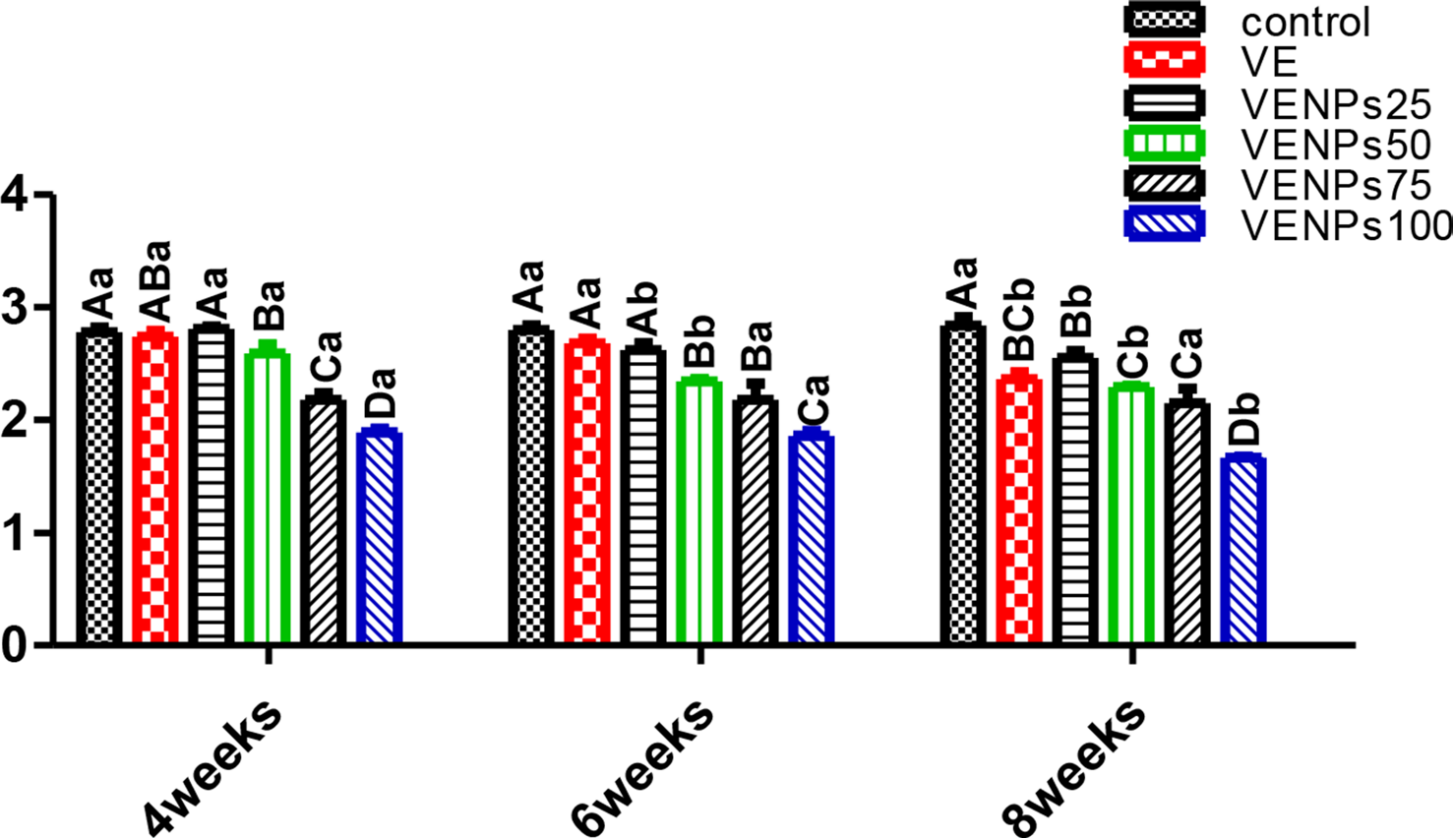
Feed conversion ratio (FCR) of the experimental Nile tilapia. Notes: VE; vitamin E, VENPs; vitamin E nanoparticles (25, 50, 20, and 100 mg/kg fish feed). Data as means (column) and standard error (small bar), values with different letters differ are significantly differed at (*P* ≤ 0.05) capital one (between the treatments in same period) small one (the same treatment in different periods)

In Fig. 6, regardless of the feeding period, all VENP groups had significantly lower FCR than the control group. Meanwhile, fish that received VENPs25 and bulks VE were insignificantly different, and the control fish had a higher FCR than all other groups. During the 8 weeks, VENPs100 was significantly the lowest, followed by VENPs75 and VENPs50, 1.67, 2.15, and 2.29, respectively.

### Innate immunity

**Fig. 7:**
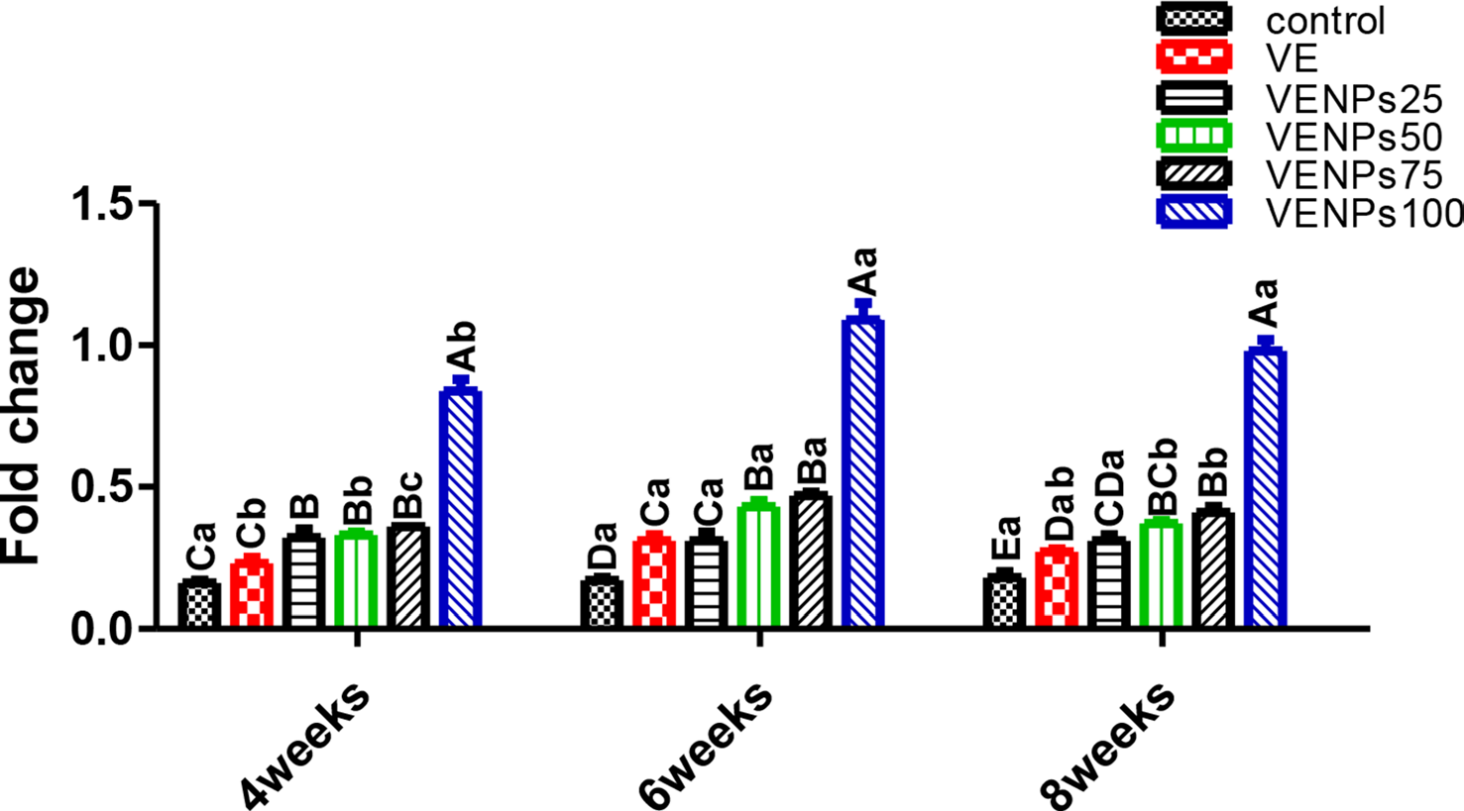
Lysozyme (U/mL) of the experimental Nile tilapia. Notes: VE; vitamin E, VENPs; vitamin E nanoparticles (25, 50, 20, and 100 mg/kg fish feed).Data represented as means ± standard error. Mean values with different capital letters at the row differ significantly at (*P* ≤ 0.05)

In Fig. 7, serum lysozyme was significantly increased in all VENPs groups in the three experimental periods. The serum of the VENPs100 group (0.84–1.09 U/mL) was upsurge LYZ (about 5 times as the control), followed by VENPs 25, 75, and 100 (about 2 times as the control) finally came with insignificant differences in VE and the control. In periods 6 weeks and 8 weeks, VE insignificantly differed from VENPs25. In both periods, 6-week and 8-week, all experimental groups were significantly higher than the control group.

**Fig. 8:**
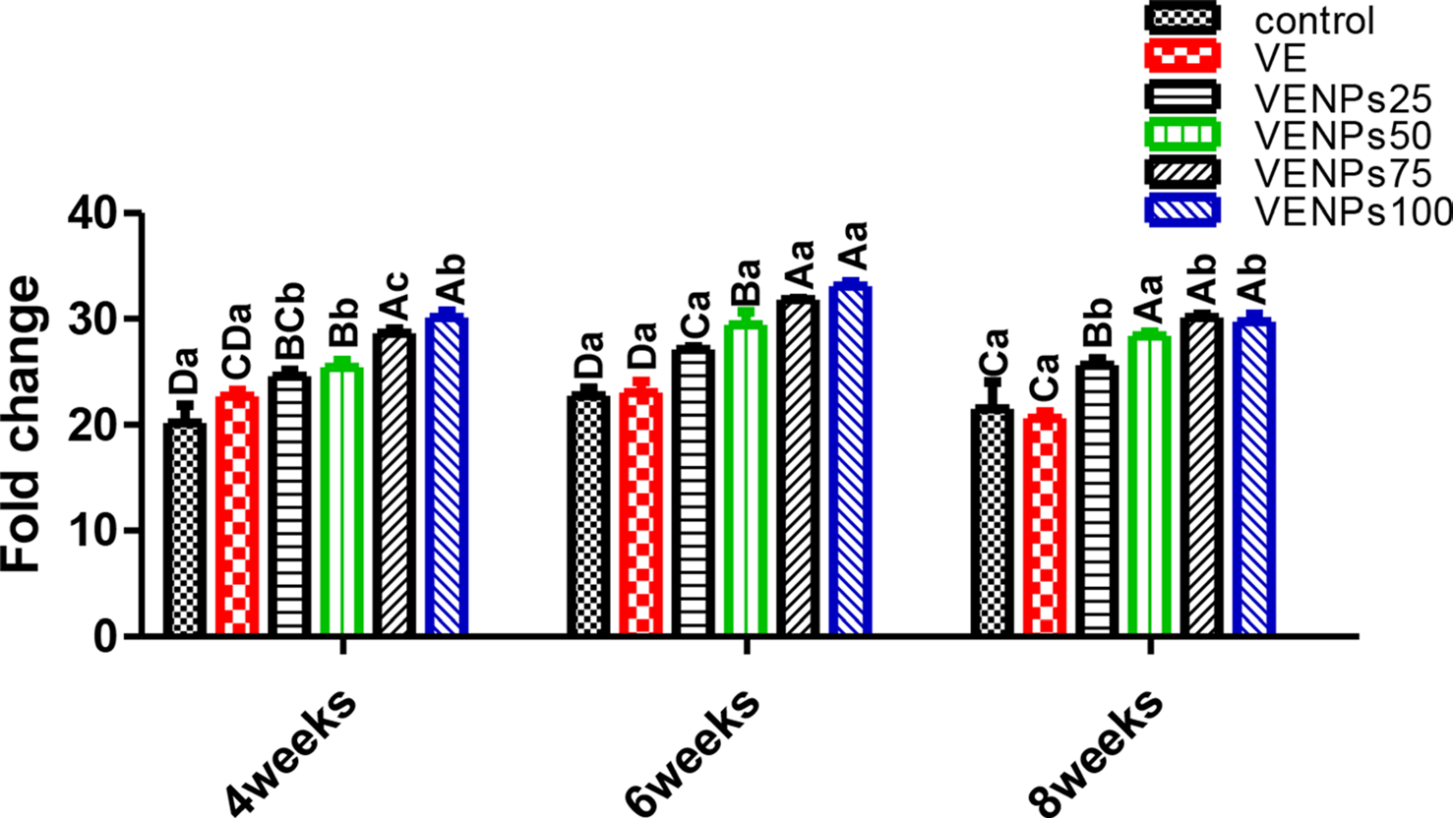
Serum antibacterial (%) of the experimental Nile tilapia. Notes: VE; vitamin E, VENPs; vitamin E nanoparticles (25, 50, 20, and 100 mg/kg fish feed). Data represented as means ± standard error. Mean values with different capital letters at the row differ significantly at (*P* ≤ 0.05)

In Fig. 8, serum antibacterial activity was gradually enhanced by increasing the VENPs supplementation. VENPs100 and VENPs75 were significantly higher, followed by VENPs50 and VENPs25 compared to the control group, 30.1%, 28.6%, 25.4%, 24.56%, 22.7, and 20.17%, respectively. The VE and VENPs25 groups were insignificantly differed (*P* ≤ 0.05). In 6-week and 8-week periods, VENPs25 was significantly higher than VE. The SAA of VENPs 50, 75, and 100 groups were insignificantly different; they were significantly higher than the other groups (*P* ≤ 0.05).

**Fig. 9:**
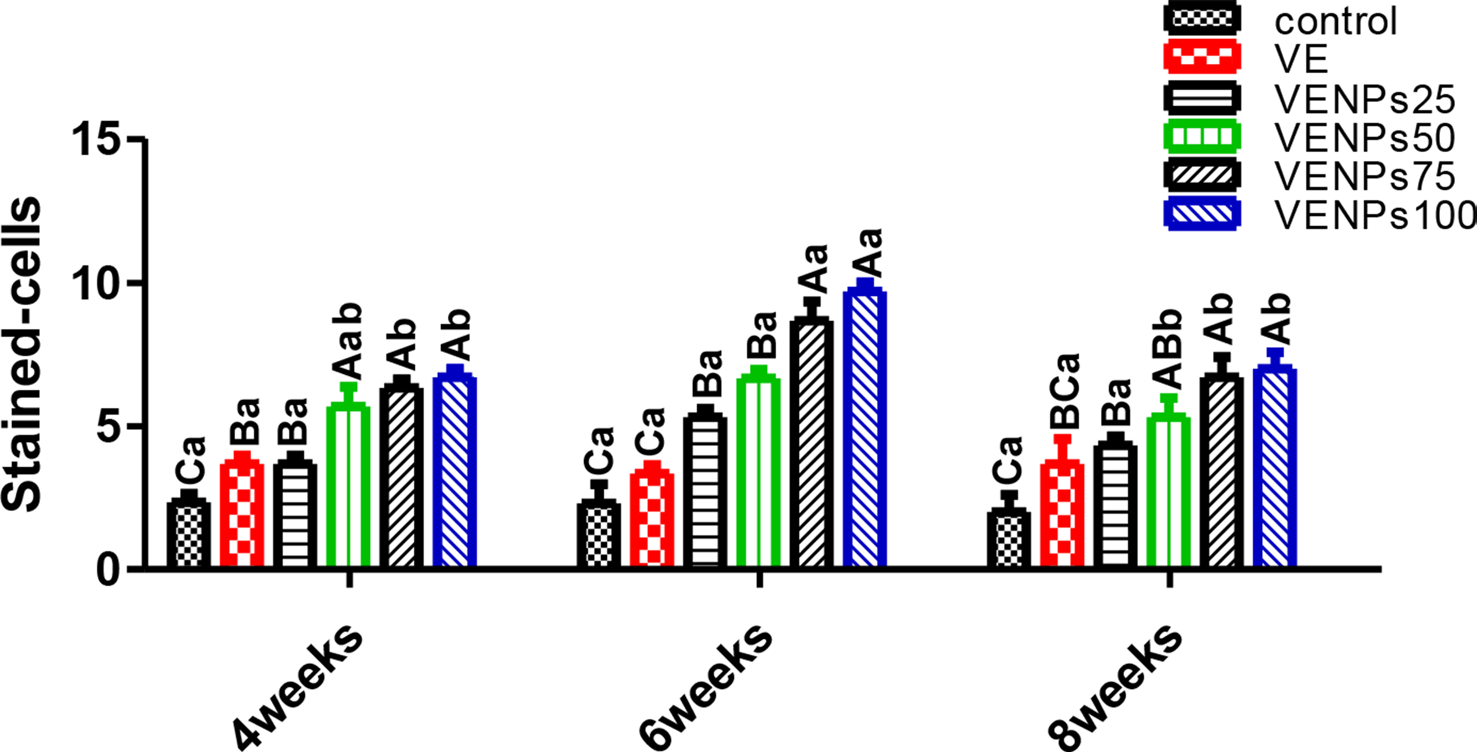
Oxidative burst activity (OBA) (stained cells) of the experimental Nile tilapia. Notes: VE; vitamin E, VENPs; vitamin E nanoparticles (25, 50, 20, and 100 mg/kg fish feed). Data as means (column) and standard error (small bar), values with different letters differ are significantly differed at (*P* ≤ 0.05) capital one (between the treatments in same period) small one (the same treatment in different periods)

In Fig. 9, The activity of heterophils of the experimental Nile tilapia was assessed by measuring the OBA. Regardless of the supplementation period, VENPs100 and VENPs75 groups had significantly higher OBA than the others (*P* ≤ 0.05); the increases were about 3 times the control. During the 6 weeks, VE and the control differed significantly. Also, VENPs25 was enhanced and insignificantly differed from VENPs50, 5.3 and 6.67 stained cells, respectively (*P* ≤ 0.05).

### Bacterial challenged experimental Nile tilapia

After each period, Nile tilapia was experimentally infected with *A. hydrophila*, MR was recorded, and the impact of VE supplementations was determined by calculating RPL. During fourteen days post-infection, the re-isolation (RI) was recorded in dead fish, and at the end, all injected fish were submitted for bacteriological investigation. It was noticed that all dead fish harbored the *A. hydrophila*, which was confirmed using the PCR technique. Fish that received dietary VENPs75 and VENPs 100 had a low MR of about 40% compared with the control, 70% (Table 1).

**Table 1:** Experimental Nile tilapia challenge with *Aeromonas hydrophila.* (*n* = 10)

Items		Control -ve	Control +ve	VE	VENPs 25%	VENPs 50%	VENPs 75%	VENPs 100%
4-week	MR %	10	**60**	60	60	60	50	40
	RLP %	-	**-**	0	0	0	16.67	33.3
	RI %	-	**100**	100	80	80	50	50
6-week	MR %	10	**60**	50	60	50	40	40
	RLP %	-	**-**	16.67	0	16.67	33.3	33.3
	RI %	-	**100**	80	80	60	50	50
8-week	MR %	10	**70**	60	60	50	40	40
	RLP %	-	**-**	14.29	14.29	28.57	42.86	42.86
	RI %	-	**100**	100	80	60	50	50

Note Control –ve; (negative), Control + ve; (positive), MR; Mortality rate, RLP; Relative protection level, RI; Re-isolation, VE; Vitamin E, VENPs; Vitamin E nanoparticles

In Table 1, Increasing the supplementation period elevated RPL% from 33.3 to 42.86% (VENPs100), 16.67–42.86% (VENPs 75), 0 to 28.57% (VENPs50), and 0 to 14.29% (VENPs25 and VE).

The recorded RI did not differ (VENPs100 and VENPs75), which was 50% regardless of the supplementation period. The RI of fish (VENPs50) decreased from 80% during 4 weeks to 60% at 6 and 8 weeks. Bacteria were re-isolated from the VE group from 80 to 100% (Table 1).

**Fig. 10:**
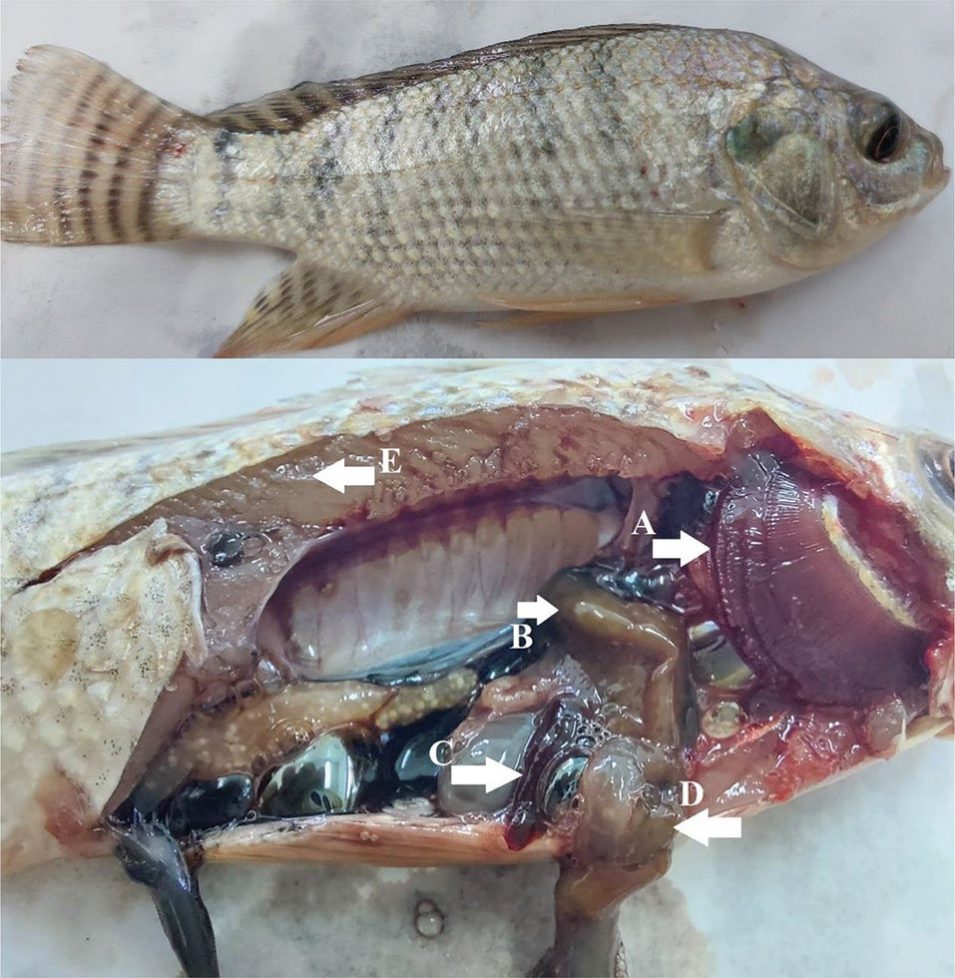
Nile tilapia fed vitamin E nanoparticles (VENPs). Clinical signs normal body coloration without any alterations, postmortem (**A**) dark reddish gills, (**B**) light brownish liver, (**C**) slight splenomegaly, (**D**) patial empty intestine, (**E**) thick musculature

**Fig. 11:**
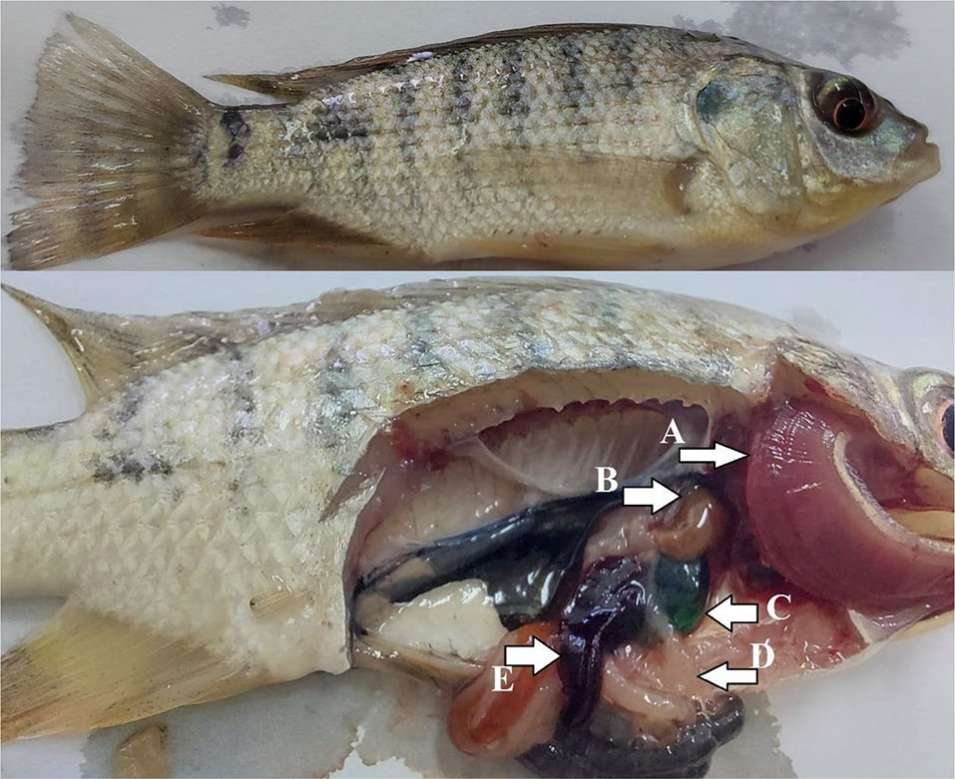
The control fish with small body size with large caudal fin, postmortem (**A**) pale gills, (**B**) light brownish liver, (**C**) ligh-greenish gall bladder, (**D**) empty intestine, and (**E**) splenomegaly

In Fig. 10, Nile tilapia fed dietary VENPs showed normal body coloration, dark reddish gills, light brownish liver, partial empty intestine, and well-nourished thick musculature. While control fish (Fig. 11) had small body sizes with pale gills. Postmortem signs were light brownish liver, light greenish gall bladder, and empty intestine.

## Discussion

In this study, the incorporation of nanocomposite VENP size was about 20 nm in Nile tilapia diets, resulting in high fish performance compared to the control fish and those who received dietary VE (bulk). Chitosan, with a size of 84.86 and 150 nm, was in-toxic to zebrafish embryos even with the high dose (150 to 400 mg/L) [37, 38]. In contrast, zebrafish embryos exposed to 40 and 30 mg/L of chitosan nanoparticles (200 and 340 nm) showed significant MR; also, the toxicity was correlated with nanoparticle concentration and exposure period, and nanoparticles with smaller size possess high toxicity [39].

In this work, Nile tilapia-fed diets contained VE (bulk form) and gradual doses (VENP25-100) for 4, 6, and 8 weeks. These diets improved the growth parameters as WG, TG, DWG, and RGR were significantly and gradually increased with high doses (VENPs50-100) regardless of the feeding periods. This achievement was accompanied by lower FCR than the control and VE groups. In accordance, Nile tilapia (14.74 ± 0.06 g) that received dietary chitosan particles (300 mg/kg) showed enhancements in their performance, which manifested by high growth rate and improved antioxidative and immune status; also, they had lower serum AST and ALT values compared to the control fish regardless rearing under different stocking densities for 70 days [40]. Similar results were reported with *Cyprinus carpio* [41]. Also, hybrid male abalone (*Haliotis fulgens* ♂. *H. discus hannai* ♀) supplemented with dietary VE (0, 0.1, 0.3, and 0.5 g/kg), fish achieved higher WG and SGR with diet containing 0.1 g VE /kg [42]. Dietary chitosan and nano-chitosan composites are considered a good growth promotor for freshwater and marinewater fishes such as gibel carp (*Carassius auratus* gibelio), rainbow trout, sea bass (*Dicentrarchus labrax*), loach (*Misgurnus anguillicaudatus*), grey mullet (*Mugil cephalus*), and Nile tilapia [43, 44]. These results could be due to the nano-sized of the composite increased bioavailability and dispersion level of dietary VE [45, 46]. Also, chitosan can activate digestive enzymes (trypsin, pepsin, alkaline phosphatase, and amylase), inhibiting the propagation of pathogenic bacteria, activating beneficial bacteria, and enhancing intestinal structure [43, 47, 48]. Another finding claimed that a diet containing chitosan nanoparticles (5 g/kg) insignificant affects silver carp growth compared to the control ones [17]. These findings may be attributed to fish species, nanoparticles (size, electric charges, crystal shape), and supplementary levels.

In this work, the immune status of the experimental Nile tilapia was tested by determining serum LYZ, SAA, and OBA; these activities were boosted after feeding VENPs-potentiated diets regardless of the feeding period. Interestingly, in these activities, 6-week and 8 weeks, all experimental groups, VENPs and VE, were significantly improved compared to the control fish, which was statistically insignificant and differed with VE fish. Parameters of innate immunity of fish that received the VENPs25 diet insignificantly differed from those with the VE diet during all periods. Similarly, Harsij et al. [49] reported that rainbow trout that received dietary VC, VE, and SeNPs had higher serum immunoglobulins and LYZ levels than the control fish. In addition, Alishahi et al. [46] found that rainbow trout supplemented with dietary chitosan loaded with VC had high serum LYZ activity. However, Naderi et al. [50] observed that dietary VE and Nano-sized Se resulted in higher LYZ activity in rainbow trout with insignificant Ig and ACH50 level alterations. In addition, dietary nano-sized Se and VE or VC are good immunostimulators; they primarily regulate pro-inflammatory cytokines [51]. Similarly, dietary chitosan nanoparticles could significantly raise the activity of LYZ in Nile tilapia serum [52] and silver carp [17]. In addition, chitosan has been described to have immune-modulating actions such as activating macrophages, encouraging cytokine release, and enlarging antibody responses [53]. These results could be the ability of chitosan to stimulate the non-specific immune responses of the fish [54].

Experimental Nile tilapia received dietary VENPs75 and VENPs100 had a low MR, about 40%, compared with the control, 70%. Increasing the supplementation period resulted in elevating RPL% from 33.3% to 42.86 (VENPs100), 16.67–42.86% (VENPs 75), 0 to 28.57% (VENPs50), and 0 to 14.29% (VENPs25 and VE). The RI was 50% in the VENPs100 and VENPs75 groups during the experimental periods. In accordance, Do-Huu et al. [55] found that the*Photobacterium damselae* counts were significantly diminished in pompano *(Trachinotus ovatus)* fish-fed diet supplemented with VE at a dose of 0.1 g/kg; also, they noticed that the survival rate was significantly enhanced even under ammonia. Similarly, VE could significantly hinder the biofilm production and intestinal colonization of *Vibrio campbellii* with an insignificant decrease in bacterial growth, resulting in a significant high SR of tomato clownfish, offering safe therapy for bacterial infection [56]. Accordingly, the immunity of *C. carpio* was improved *by dietary* chitosan nanoparticles and VE supplementation, fish could resist experimental *A. sobria* infection increasing their SR [57]. Similarly, rohu *(Labeo rohita*) fingerlings supplemented with dietary chitosan nanoparticles showed a low MR% and high SR% after being challenged with the pathogenic strain of *A. hydrophila* [3], also *O. niloticus* showed similar results after the challenge with *A. sobria* [58]. Likewise, Kumar et al. [59] found that short-term (20 days) application of dietary chitosan nanoparticles (0.0, 0.25, 0.50, 1, and 2 g/kg fish feed), OBA, LYZ, myeloperoxidase, and catalase activity were significantly improved with high survival rate in rohu fish received 1 g/kg fish feed and challenged against pathogenic *A. hydrophila*. These results could be due to the ability of chitosan nanoparticles to penetrate the bacterial cell wall, resulting in the leakage of cell fluids and the immunostimulant and antioxidant properties [60, 61].

## Conclusion

Dietary nanoparticles of Vitamin E (100 mg), protected with nano-chitosan, for 4, 6, and eight weeks could enhance the performance of Nile tilapia (70.85 ± 0.2 g). the growth parameters FW, TG, DWG, and RGR were linearly gradually increased after receiving different dietary levels of VENPs 25%, 50%, 75%, and 100% of the recommended dose (100 mg/kg fish feed), regardless of the incorporation levels, they achieved significant low FCR values than the control fish and those received VE (bulk form). Immune activities (LYZ, SAA, and OBA) were improved in fish fed the diet containing VENPs. Dietary VENPs could achieve high RPL%, making fish less vulnerable to infectious bacteria.

## Data Availability

No datasets were generated or analysed during the current study.
